# DangerTrack: A scoring system to detect difficult-to-assess regions

**DOI:** 10.12688/f1000research.11254.1

**Published:** 2017-04-07

**Authors:** Igor Dolgalev, Fritz Sedlazeck, Ben Busby

**Affiliations:** 1 New York University School of Medicine, New York, NY, 10016, USA; 2 Department of Computer Science, Johns Hopkins University, Baltimore, MD, 21202, USA; 3 National Center for Biotechnology Information (NCBI), National Library of Medicine, National Institutes of Health, Bethesda, MD, 20894, USA

**Keywords:** Breakpoint, Structural Variants, SNP, CNV, Clinical Genetics

## Abstract

Over recent years, multiple groups have shown that a large number of structural variants, repeats, or problems with the underlying genome assembly have dramatic effects on the mapping, calling, and overall reliability of single nucleotide polymorphism calls. This project endeavored to develop an easy-to-use track for looking at structural variant and repeat regions. This track, DangerTrack, can be displayed alongside the existing Genome Reference Consortium assembly tracks to warn clinicians and biologists when variants of interest may be incorrectly called, of dubious quality, or on an insertion or copy number expansion. While mapping and variant calling can be automated, it is our opinion that when these regions are of interest to a particular clinical or research group, they warrant a careful examination, potentially involving localized reassembly. DangerTrack is available at https://github.com/DCGenomics/DangerTrack.

## Introduction

The advent of next generation sequencing has enabled the comparison of cells, organisms, and even populations at the genomic level. Whole genome sequencing experiments are run worldwide on a daily basis with various aims, from exploring novel genomes to diagnosing complex variations in high-ploidy cancer samples. A common step in all of these studies is the mapping of the sequence to a reference genome or assembly to identify variations (whole genome sequencing) or expression (RNA sequencing) of the sample.

Multiple studies so far have suffered from mapping artifacts typically occurring in highly variable regions, including single nucleotide polyporphisms (SNPs) and structural variants (SVs), which may be repetitive regions or regions that are not correctly represented by the reference genome ( Degner *et al*., 2009). Multiple methods have been suggested to overcome this bias, including constructing a personalized reference genome ( Satya *et al.*, 2012), sequencing the parental genomes ( Graze *et al.*, 2012), building graph genomes over all known variants ( Dilthey *et al.*, 2015), or carefully reconciling particular subregions. The latter includes discarding reads using a mapping quality filter, realigning reads locally, or computing a localized *de novo* assembly using the Genome Analysis Toolkit to improve the quality of SNP calls. However, all these methods often depend on the sample quality (e.g. coverage, error rate), may result in additional expenses, and are often optimized only for human genome data.

Here, we present DangerTrack, the first approach to automatically classify difficult-to-assess regions by combining annotated features, such as mappability and SV calls. DangerTrack can be applied to any genome and organism of interest. It runs within minutes and provides a Browser Extensible Data (BED) file with a score for every 5 kb region. The height of the score indicates the trustworthiness of the region in terms of SNP calling, and thus how difficult an accurate mapping can be. DangerTrack represents a flexible and easy to use method to detect hard-to-analyze regions with a pure mapping approach. We compared the results of DangerTrack to the blacklisted regions of ENCODE ( https://personal.broadinstitute.org/anshul/projects/encode/rawdata/blacklists/hg19-blacklist-README.pdf), as well as to the list of problematic regions from the National Center for Biotechnology Information (NCBI).

## Methods

### Incorporation of structural variation data sets

We downloaded the SVs dataset from the 1000 Genomes Project ( Sudmant *et al.*, 2015) (1KG) from dbVar (estd219; https://www.ncbi.nlm.nih.gov/dbvar/studies/estd219/), as well as a 16-candidate SV callset from the Genome in a Bottle (GIAB; Zook *et al.*, 2016) (Ashkenazi son dataset available at ftp://ftp-trace.ncbi.nlm.nih.gov/giab/ftp/release/AshkenazimTrio/HG002_NA24385_son/latest; NIST Reference Material 8391: HG002 and NA24385). These Variant Call Format (VCF) datasets were converted into BED files using SURVIVOR ( Jeffares *et al.*, 2017) (available from https://github.com/fritzsedlazeck/SURVIVOR). Each SV was represented by two entries in the BED file, listing the breakpoints of each reported SV.

Next, we binned the breakpoints in 5 kb windows and counted the number of SVs in these windows. The number of SV breakpoints per window was normalized by the 99% quantile number of breakpoints within a window across the whole genome. Thus, the higher the ratio, the more SV breakpoints are in a given window, and therefore the less trustworthy the reference seems to be.

### Incorporation of mappability tracks

We downloaded the 50 bp and 100 bp mappability tracks from UCSC ( http://hgdownload.cse.ucsc.edu/goldenPath/hg19/encodeDCC/wgEncodeMapability/).

These mappability tracks contain a measurement for each base of the reference genome. These tracks were generated using different window sizes, with high signals indicating areas where the sequence is unique. The GEM (GEnome Multitool) mapper ( http://big.crg.cat/services/gem_aligner) was used to generate CRG k-mer alignability. The method is equivalent to mapping sliding windows of k-mers back to the genome. For each window, a mappability score is computed as 1 divided by the number of matches found in the genome. Thus, a score of 1 indicates one match in the genome, 0.5 indicates two matches in the genome, and so on.

Next, we computed the score for uniqueness of regions. This was done by subtracting the average mappability value from 1. Thus, a value of 0 represents a unique region. Similarly to the SVs computation method, we summarized the average uniqueness score per 5 kb window, obtained by simple average across the window for both 50 bp and 100 bp tracks.

### DangerTrack score calculation

We computed the DangerTrack score by combining all four features with a uniform weighting schema. Note that our score operates between 0 and 1, where 0 means a unique, easy-to-assess region, and 1 means a region that is repetitive and enriched for structural variations.

The resulting genome-wide DangerTrack score in BED and bedGraph formats are available at: https://github.com/DCGenomics/DangerTrack. The repository also contains the bash and R scripts for downloading, cleaning, and summarizing the data, so the score can be computed independently or for different window sizes. The code can be adapted for use with other genomes assuming comparable mappability and structural variation data sets are available.

### Comparison to blacklist regions from NCBI and ENCODE

We downloaded the Blacklisted Regions that are defined as problematic by ENCODE Data Analysis Center ( https://www.encodeproject.org/annotations/ENCSR636HFF/), as well as the list from the Genome Reference Consortium ( ftp.ncbi.nlm.nih.gov/pub/grc/human/GRC/Issue_Mapping/) of regions that either underwent manual curation from GRch37 to GRch38 or are listed as problematic for future versions of the human genome. For the comparison, we binned the list of regions similar to our approach in 5 kb regions. Next, we compared the values between our track and the generated ENCODE and GRC tracks.

## Results

### Data exploration

**Figure 1.**
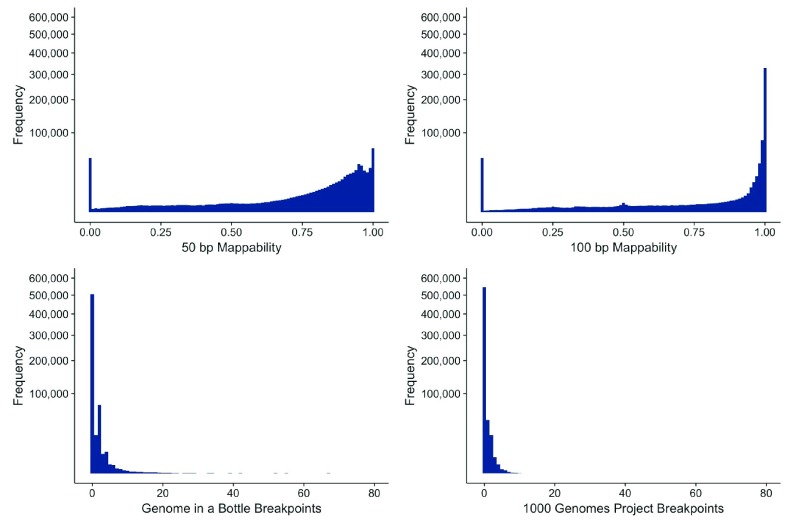
Distributions of the observed frequencies over the binned datasets. (A) Histogram over the hg19 genome of mappability with respect to 50 bp. (B) Histogram over the hg19 genome of mappability with respect to 100 bp. (A and B) are obviously closely related, with the exception that (A) (50 bp regions) includes more regions that have on average a lower score. (C) Distribution of SVs across the hg19 genome based on 16 SV data sets. (D) Distribution of SVs across the hg19 genome based on the 1000 Genomes Project call set.

To assess the ability of DangerTrack to highlight suspicious regions, we computed the DangerTrack score over the human reference genome (hg19) using data from the 1000 Genomes Project and GIAB, as well as mappability tracks from UCSC. We downloaded 72,432 SVs from the 1000 Genomes Project data and 135,653 SVs from the GIAB database, for a total of 363,234 breakpoints. Figure 1 shows the histogram of the number of SV breakpoints within each 5 kb bin. As expected, the SV events predicted by the 1000 Genomes Project (labeled 1KG in Figure 1) and GIAB data highlight regions in the genome with high structural variability. However, very few regions exist that incorporate more than 20 events within 5 kb. Interestingly, these two tracks are not very similar, with a correlation of only 0.06 over a subsample of 10,000 bins.

For the mappability data, we naturally expect a high correlation, since the regions that are not unique within a 100 bp region will also not be unique given a 50 bp sequence. The correlation over the 10,000 subsampled 5 kb regions is therefore high (0.95). We chose these two mappability tracks as they reassemble the often-used read length and also take into account local alignment-based clipping of reads.

### Evaluation of DangerTrack

**Figure 2.**
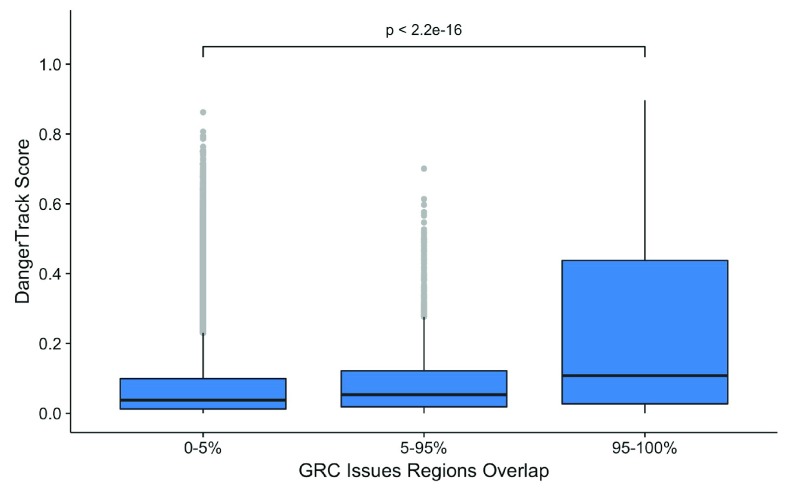
Comparison between DangerTrack score and GRC updated regions or regions that are still manually vetted.

**Figure 3.**
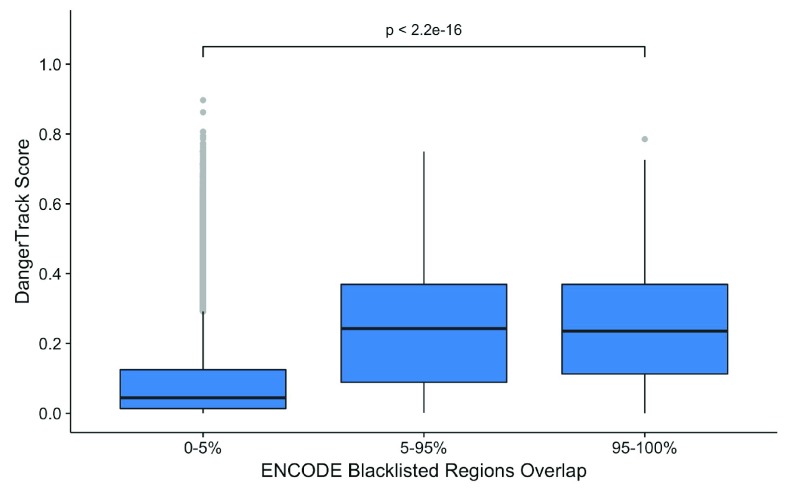
Comparison between DangerTrack score and ENCODE blacklisted regions.

**Figure 4.**
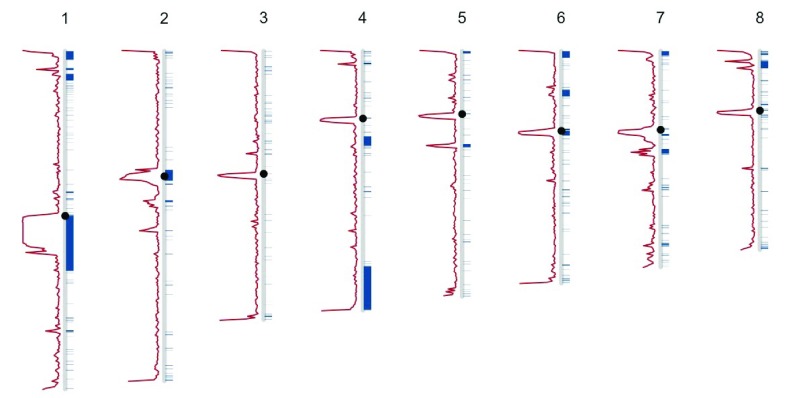
Comparison of DangerTrack score (red) and known blacklisted regions of ENCODE and GRC (blue) along chromosomes 1–8.

Next, we compared the DangerTrack score to manually curated regions from ENCODE and NCBI. These regions represent areas along the genome that are either discarded due to their problematic mapping from previous experiences during the ENCODE project, updated in GRCh38, or still under manual curation for future genome releases. Figure 2 and Figure 3 represent the comparison between the DangerTrack score and the listed regions for ENCODE and NCBI, respectively. We observe a very high correlation for both tracks, highlighting that the DangerTrack score captures these regions. Figure 4 represents the overlap of the DangerTrack score and the annotated regions from Encode and NCBI for chromosomes 1–8.

## Conclusions and next steps

The results of DangerTrack overlap with previously-established troubling regions from the ENCODE blacklist and with regions of assembly error identified by the Genome Reference Consortium. Furthermore, we identified 48,891 5 kb regions (7.9% of all regions) that are not trustworthy. Thus, the mappability score and the concentration of SV breakpoints in a region indicate that the region is less reliable for SNP calling alone. This difficulty may be due to a high degree of difference in the reference sequence or the number of unresolved regions. While we showed that DangerTrack is capable of capturing these challenges for hg19, this method is universally applicable regardless of organism. The mappability tracks can be established easily and SV calls from other organisms can be incorporated. Nevertheless, DangerTrack is only a first step in understanding the underlying complexity of certain regions. Future work will include a revised weighting of the individual tracks.

## Software availability

The code for the pipeline and the resulting genome-wide DangerTrack score are publically available at: https://github.com/DCGenomics/DangerTrack

Archived source code as at time of publication: doi, 10.5281/zenodo.438344 ( igor & DCGenomics, 2017).

License: MIT
