# Supplementary material for: DangerTrack: A scoring system to detect difficult-to-assess regions
Source: F1000Res. 2017 Apr 7;6:443. [Version 1] doi: 10.12688/f1000research.11254.1 (PMC5405793; doi:10.12688/f1000research.11254.1)
Supplement: Peer review history

# Peer review: Referee response for version 1

**DOI:** 10.5256/f1000research.12141.r21627

**Published:** 2017-04-24

**Authors:** Andrew Carroll

**Affiliations:** 1 DNAnexus, Mountain View, CA, USA

This work uses the frequency of detected structural variant events as a proxy for reference quality and thus as a measure of trustworthiness for variant calls in a region. The foundation for the work is a consensus set of multiple SV methods applied in both Genome in a Bottle and 1000 genomes.

 The concept is interesting and well applied. The analysis is clearly communicated, and the code is both openly available and interpret-able.

 I do have a minor issue with the communication of the issue. The article doesn't get specific about why performing analysis in these genomic regions might be "dangerous", which I would categorize as:

1. Regions which represent a problem in the reference itself (either regions of mis-assembly, or locations where a rare SV event was present in the sequence used which the rest of the population doesn't have)
2. Regions which may contain "SV hotspots" 
3. Low complexity regions, centromeric regions, telomeric regions.
4. Segmental duplications with insufficient divergence, mobile elements.
5. Regions of high heterozygosity in the population - which may or may not be SV in origin but due to their diversity may manifest as SV events.

Since the paper doesn't specifically break down these possibilities, I feel that the tone of the discussion would imply to a naive reader that these regions are "dangerous" because they are SV hotspots, when I think the authors would agree with me that reference assembly issues, low complexity regions, and segmental duplications are probably responsible for most of the "dangerous" regions.

 It might be worthwhile to spend a few sentences to explain why a region might be dangerous, though the paper is written so tightly around the problem that it might distract the paper from its core point.

 Separately, another distinction it might be worthwhile to make is that the SVs from the 1000 genomes project are quite different than the HG002 SVs. In one case, we have SVs generated over a population using more limited sequencing technology. HG002 is very different, where we are instead generating a wealth of calls from a single sample. Both approaches are worthwhile and it is appropriate to combine them. It would be interesting to note whether there are differences between the types of regions the two approaches identify.

 From this point, one could ask a number of additional questions beyond the scope of the manuscript. For example - which of these regions are no longer SV hotspots when using longer reads.

 Hopefully authors of SV tools will begin to take this DangerTrack into account when developing their methods - either to give quality values to their calls, or as a means to separate calling on hard and easy regions of the genome.

 Overall, the work is sound and the paper very well written.

I have read this submission. I believe that I have an appropriate level of expertise to confirm that it is of an acceptable scientific standard.

# Peer review: Referee response for version 1

**DOI:** 10.5256/f1000research.12141.r21624

**Published:** 2017-04-24

**Authors:** Justin M. Zook

**Affiliations:** 1 Material Measurement Laboratory, National Institute of Standards and Technology (NIST), Gaithersburg, MD, USA

The authors present a tool that finds potentially problematic regions for small variant calling, based on mappability and previously called SVs. It's an interesting and useful tool, and I have a few suggestions for improvement:

1. It is worth noting that the 1000 Genomes SVs were discovered using only short reads in thousands of individuals, and the GIAB SVs were discovered using short and long reads in only one mother-father-son trio. There are nuances to this that would be useful to discuss. In particular, GIAB SV locations may or may not be SVs in other individuals, and because they are not highly curated, they may contain FPs around repetitive regions, but these are still good regions to identify as problematic for small variant calling
2. I don't see the script for running SURVIVOR commands and generating the dangertrack bed files in the GitHub site, which would be useful to include to reproduce the results.
3. When the authors say "Furthermore, we identified 48,891 5 kb regions (7.9% of all regions) that are not trustworthy," what dangertrack score threshold do they use (e.g., ==1, >0.9, >0.5, ...)?
4. It may be interesting to compare to the GIAB high-confidence bed file for NA12878 ( ftp://ftp-trace.ncbi.nlm.nih.gov/giab/ftp/release/NA12878_HG001/NISTv3.3.2/GRCh37/HG001_GRCh37_GIAB_highconf_CG-IllFB-IllGATKHC-Ion-10X-SOLID_CHROM1-X_v.3.3.2_highconf_nosomaticdel.bed) and the Platinum Genomes bed file for NA12878 ( ftp://ftp-trace.ncbi.nlm.nih.gov/giab/ftp/technical/platinum_genomes/2016-1.0/hg19/small_variants/ConfidentRegions.bed.gz), since these are both regions where they purport to make high-confidence small variant calls.

I have read this submission. I believe that I have an appropriate level of expertise to confirm that it is of an acceptable scientific standard.

# Peer review: Referee response for version 1

**DOI:** 10.5256/f1000research.12141.r21625

**Published:** 2017-04-24

**Authors:** Melissa A. Gymrek, Nima Mousavi

**Affiliations:** 1 Department of Medicine, University of California, San Diego, La Jolla, CA, USA; 2 Department of Computer Science and Engineering, University of California, La Jolla, CA, USA; 3 Department of Electrical and Computer Engineering, University of California, La Jolla, CA, USA

The authors present DangerTrack, a method to score regions of the genome that are likely to be problematic for variant calling. The method combines information from structural variant catalogs and mappability scores across the genome into a single track whose score is meant to correlate with the “trustworthiness” of a region.

 Assessing which regions of the genome are likely to be error-prone for variant calling is indeed an important issue, especially for the use case mentioned of clinicians and biologists interested in particular variants. The manuscript is for the most part well-written and the method is clearly described. However, the rationale for developing a new annotation track on top of existing annotations such as the ENCODE “black list” is not well described, and the authors do not provide sufficient evidence of the claim that DangerTrack successfully classifies “difficult” regions of the genome for variant calling. These and other concerns are outlined in more detail below.

**Major comments:**

- The rationale and goal are not clearly defined: What was the primary rationale for developing DangerTrack? It was not immediately clear why another annotation is needed, given that tracks such as the ENCODE blacklist and the NCBI problematic region list already exist. One potential reason is that those lists are inadequate, and we need a list that is better at picking out truly problematic regions for variant calling. If that was indeed the goal, the authors do not present sufficient evidence that their annotation is any better than the existing lists. On the other hand, another reasonable motivation to create this tool is that the ENCODE/NCBI lists were created manually, and could not be easily constructed for a new genome or a new individual. If that is the case, the authors should explicitly state early on that this was their primary motivation. Finally, there are other automated tools/tracks such as RepeatMasker and dustmasker that might be used to filter likely low quality variants. How does DangerTrack compare to those?
- Insufficient evaluation: The authors claim that their score, based on the # SV breakpoints/5kb, tracks with SNP call quality. However, this is never backed up with any evidence. Thus, it is impossible to tell whether this track actually adds any value in filtering low quality SNP calls. One potential validation would be to look at SNP quality scores or SNP call accuracy stratified by DangerTrack value, and show a relationship. If DangerTrack does a better job of classifying incorrect vs. true SNP calls than other tracks, then that would be clear evidence that it gives value added over existing tools. Similarly the discussion states that the authors identify ~48K “untrustworthy” regions, but there is no data to back up the statement that those regions are indeed enriched for incorrect calls.

**Minor comments:**

- Last paragraph of introduction, suggest to change “height of the score” to “magnitude of the score”
- Last sentence before “Evaluation of DangerTrack”, change “reassemble” to “resemble”
- Same sentence, how are the mappability tracks related to base-clipped reads?
- How did the authors decide on the weighting scheme to combine different features?
- The low overlap between 1000 Genomes and GIAB breakpoints raises concerns over how reproducible DangerTrack will be and how sensitive it is to the quality of the SV catalog used as input.
- Figures 2 and 3 are not well described, it was not clear what is being depicted.
- The authors indicate that there is “very high correlation” with the ENCODE blacklist track. This should be stated more quantitatively, in terms of e.g. correlation or % overlap.

We have read this submission. We believe that we have an appropriate level of expertise to confirm that it is of an acceptable scientific standard, however we have significant reservations, as outlined above.
